# M2 Polarization and Inhibition of Host Cell Glycolysis Contributes Intracellular Survival of *Salmonella* Strains in Chicken Macrophage HD-11 Cells

**DOI:** 10.3390/microorganisms11071838

**Published:** 2023-07-19

**Authors:** Haiqi He, Kenneth J. Genovese, Ryan J. Arsenault, Christina L. Swaggerty, Casey N. Johnson, J. Allen Byrd, Michael H. Kogut

**Affiliations:** 1 Southern Plains Agricultural Research Center, USDA-ARS, College Station, TX 77845, USA; 2 Department of Animal and Food Sciences, University of Delaware, Newark, DE 19716, USA

**Keywords:** chicken macrophage cell, foodborne pathogens, *Salmonella*, glycolysis, macrophage polarization, nitric oxide, *Salmonella* intracellular survival

## Abstract

Salmonella *enterica* is a group of facultative, gram-negative bacteria. Recently, new evidence indicated that *Salmonella* could reprogram the host metabolism to increase energy or metabolites available for intracellular replication. In this study, using a chicken-specific kinomic immunometabolism peptide array analysis, we found that infection by *S.* Enteritidis induced significant phosphorylation changes in many key proteins of the glycolytic pathway in chicken macrophage HD-11 cells, indicating a shift in glycolysis caused by *Salmonella* infection. Nitric oxide production and changes of glycolysis and mitochondrial oxidative phosphorylation (OXPHOS) represented by extracellular acidification rate (ECAR) and oxygen consumption rate (OCR), respectively, were measured in chicken macrophages infected with three *Salmonella* strains (*S*. Enteritidis, *S.* Heidelberg, and *S*. Senftenberg). The infection reduced glycolysis and enhanced OXPHOS in chicken macrophages as indicated by changes of ECAR and OCR. *Salmonella* strains differentially affected macrophage polarization and glycolysis. Among three strains tested, *S*. Enteritidis was most effective in downregulating glycolysis and promoting M2 polarization as measured by ECAR, ORC, and NO production; while *S*. Senftenberg did not alter glycolysis and may promote M1 polarization. Our results suggested that downregulation of host cell glycolysis and increase of M2 polarization of macrophages may contribute to increased intracellular survival of *S.* Enteritidis.

## 1. Introduction

*Salmonella enterica* is a group of facultative, gram-negative bacteria ranging from self-limiting gastroenteritis (non-typhoidal *Salmonella*) to life-threatening typhoid fever (serovar Typhi) in humans [1]. More than 2500 serovars have been identified, most of them are non-typhoidal *Salmonella* and are highly adaptive to divergent environments and commonly reside in the intestinal tracts of many animals. *Salmonella* from contaminated meats, poultry, and eggs is a leading cause of foodborne illnesses in the US [2]. In contrast to humans, chickens infected with non-host specific *Salmonella* serovars largely display no symptoms [3].

In chickens, *Salmonella* that cross the intestinal barrier are taken up by polymorphonuclear heterophils and macrophages. The phagocytized *Salmonella* are effectively killed by heterophils [4]; however, intra-macrophage *Salmonella* can survive, despite chicken macrophages being capable of producing various bactericidal substances, including reactive radical oxygen species (ROS), nitric oxide (NO), lysozyme, and proteolytic enzymes when exposed to *Salmonella* [3,5]. It is well established that *Salmonella* evade macrophage killing mechanisms via the Type III Secretion System (T3SS) which secretes and delivers a repertoire of virulence effector proteins into host cells to facilitate invasion, survival, and replication inside phagocytes [6].

More recently, *Salmonella* has been found to not only alter host cellular structures and immune response, but also reprogram the host central carbon metabolism to increase host cell derived energy or metabolites available for survival and replication [7,8]. Infections by many intracellular pathogens (*Mycobacterium tuberculosis*, *brucella abortus*, *Helicobacter pylori*, *chlamydia trachomatis*, and *S. typhimurium*) have been found to drive host cell metabolic shift in the manner described as “Warburg metabolism”, characterized as increasing aerobic glycolysis rather than oxidative phosphorylation for energy and metabolites [9]. This phenomenon appears to correlate with classically activated or M1 state macrophages, in which aerobic glycolysis is elevated, resulting in increased production of lactate and Krebs cycle metabolites [10]. Glucose is the major carbohydrate which supports the glycolytic metabolism in *Salmonella* and is required for successful intracellular replication in macrophages [11]. However, several recent studies indicate that the effect of intracellular *Salmonella* on carbon metabolism in macrophages is more complex and not wholly consistent, indicating that the outcome may be influenced by both *Salmonella* strains and macrophages under investigation. In a study using human monocyte-derived macrophages, *S*. Typhi infection was found to induce a Warburg-like effect with increased glycolysis and glucose availability for intracellular replication [12]. Similarly, *S. Typhimurium* infection in mouse peritoneal macrophages and RAW264.7 cell line was found to enhance glycolysis, but intracellular *Salmonella* was found to increasingly use glycolysis intermediates, 2-, or 3-phosphoglycerate and phosphoenolpyruvate, as carbon sources when intracellular glucose was limited [13]. However, in other studies, it was found that infection with *S. Typhimurium* reduced host cell glycolysis, which leads to an impairment of phagosome maturation and clearance of intracellular bacteria in bone marrow-derived macrophages [14].

Information on immunometabolic changes during *Salmonella* infection in chickens has recently emerged, albeit rather limited. Studies have found that *Salmonella* infection induced significant phosphorylation changes in proteins involved in immune and metabolic pathways in both in vivo tissues [15,16] and in vitro chicken macrophages cell HD-11 [17] using a chicken-specific kinomic immunometabolism peptide array analysis. In these studies, *Salmonella* infection was shown to cause great perturbance in the glycolytic pathway. In a previous study, we have shown that *Salmonella* strains interact with chicken macrophages differently, resulting in significantly different outcomes in terms of intracellular survival and host cell immune response [18]. In the present study, glycolysis of chicken macrophages infected with different *Salmonella* strains and a possible association between glycolysis and intracellular survival of *Salmonella* were investigated.

## 2. Materials and Methods

### 2.1. Reagents

Cell culture medium and other products used in this study were purchased from Sigma-Aldrich (St. Louis, MO, USA) unless otherwise indicated.

### 2.2. Cell Line

The MC29 virus-transformed chicken macrophage cell line HD-11 [19] was maintained in a complete Dulbecco’s Modified Eagles Medium (DMEM) containing 10% chicken serum, antibiotics (100 U penicillin/mL and 100 µg streptomycin/mL), and 1.5 mM L-glutamine at 39 °C, 5% CO_2_, and 95% humidity. Aliquots of cell suspension (2 × 10^6^ cells/mL) were seeded into each well at 1 mL/well for 12-well plate (BD, Franklin Lakes, NJ) and allowed to grow to about 85% confluence (~36 h) before being used for infection.

### 2.3. Bacterium

*Salmonella* Enteritidis, *S.* Heidelberg, and S. Senftenberg used in the present study were initially field isolates from poultry farms and were serotyped by the National Veterinary Services Laboratory (Ames, IA, USA). These isolates were selected to resist carbenicillin-novobiocin (C-N) and have been used in our previous studies [18]. *Salmonella* stock aliquots were cultured overnight at 39 °C in BD’s TSB (Tryptic Soy Broth), the overnight cultures were diluted at 1:10 into fresh TSB and cultured at 39 °C for 4 h to reach exponential growth phase, and the bacteria were collected by centrifugation, washed, and resuspended in PBS at a final concentration of ~1 × 10^9^ (cfu, colony-forming unit)/mL, determined by colony counts on BD’s Difco’s xylose-lysine tergitol 4 (XLT4) agar plates containing C-N. Heat-killed *S*. Enteritidis (HKSE) was prepared by incubating the bacterial suspension in a 75 °C water bath for 15 min and verified by overnight culture.

### 2.4. Cell Infection with Salmonella

Culture medium was removed from the HD-11 cells and infected with 500 μL of *Salmonella* suspensions (~5 × 10^8^ cfu/mL in plain DMEM) added to each well with a multiplicity of infection (MOI) at about 50:1, in addition to three replicate wells for each serovar and incubated for 1 h at 39 °C in a 5% CO_2_ humidified incubator. At 1 h post infection (hpi), the infection medium was removed, and the cells were washed once with plain DMEM, treated with 100 μg/mL of gentamicin sulfate for 30 min to kill extracellular bacteria, and then replaced with fresh complete DMEM containing 25 μg/mL of gentamicin sulfate.

Intracellular viable *Salmonella* were determined at 2 and 20 hpi as described previously [18]. Briefly, infected cells were washed twice with PBS and lysed for 10 min in 1% Triton X-100 (in PBS). Serial 1:10 dilutions of the lysates were plated onto XLT4 agar plates containing C and N and incubated at 39 °C for 24 h. Colonies were counted to determine the cfu of intracellular viable bacteria.

### 2.5. Peptide Array Protein Phosphorylation Analysis of S. Enteritidis Infected HD-11 Cells

Peptide arrays were made by JPT Peptide Technologies (Berlin, Germany) which contain 771 unique chicken kinase substrate target peptide sequences, derived from the phosphorylation sites of 572 proteins that were printed in replicate 9 times [20]. *S.* Enteritidis infected cells, in two replicates for each time point, were collected at 1.5, 3, and 7 hpi and stored at −80 °C. Sample preparation and array analysis were performed as described previously [17,21].

### 2.6. Extracellular Acidification Rate (ECAR) and Oxygen Consumption Rate (OCR) of HD-11 Cells

Real-time and live cell analysis of glycolysis based extracellular acidification rate (ECAR) and mitochondrial oxidative phosphorylation (OXPHOS) based on the oxygen consumption rate (OCR) were performed by the Seahorse XFp Analyzer (Agilent Technologies, Santa Clara, CA, USA) in non-buffered Seahorse XF medium under basal conditions, per the manufacturer’s instruction. Aliquots of 100 µL cell suspension (2 × 10^6^ cells/mL) were seeded into each well and allowed to grow overnight and then infected with 50 µL of *Salmonella* suspensions (as described above). Extracellular bacteria were killed with 100 μg/mL of gentamicin sulfate for 30 min and the medium was replaced with Seahorse XF medium for measurement of ECAR and OCR. Three strains of *Salmonella* (*S*. Enteritidis, *S.* Heidelberg, and *S*. Senftenberg) were tested.

### 2.7. Nitrite Assay

Nitrite, a stable metabolite of NO, produced by activated macrophages, was measured by the Greiss assay [22]. Cells in 24-well plates were stimulated with 0.1 µg/mL of lipopolysaccharide (LPS) or infected with *Salmonella* strains as described above for 20 h at 39 °C in a 5% CO_2_ humidified incubator. After 20 hpi with *Salmonella* infection or LPS stimulation, aliquots of 100 μL culture supernatant from each well were transferred to the wells of a new flat-bottom 96-well plate and mixed with 50 μL of 1% sulfanilamide and 50 µL of 0.1% naphthylenediamine (both were prepared in 2.5% phosphoric acid solution) sequentially. The optical density (OD_550_) of each well was measured using a microplate reader (Molecular Devices, Sunnyvale, CA, USA). Sodium nitrite was used as a standard to determine nitrite concentrations in the cell-free medium.

### 2.8. Data Analysis

Three independent experiments were conducted to determine *Salmonella* intracellular survival and NO production. Within each experiment, three replicates were measured. Data were analyzed by One Way ANOVA followed by multiple comparisons (Tukey test) using SigmaPlot 12.0^®^ software (SYSTAT, Palo Alto, CA, USA).

## 3. Results and Discussion

### 3.1. S. Enteritidis Infection Induces Significant Phosphorylation Changes in Many Enzymes of the Glycolysis Pathway in Chicken Macrophage Cells

Survival inside the macrophage is essential for *Salmonella* virulence and systemic infection [23,24]. *Salmonella* virulence depends largely on the type III secretion system (T3SS), which secrets and delivers over 40 different virulence effectors into host cells to facilitate invading, surviving, and replicating [6]. Recent evidence further indicates that intracellular *Salmonella* highjack the host cell glycolytic pathway to acquire host cell derived energy and glucose or its intermediate metabolites for replication [12,13,14] and meanwhile deprive the host cells of energy and nutrients needed for normal cell function and immune response.

Protein kinases and phosphatases control protein phosphorylation and regulate metabolic pathways and cellular processes involved in nearly every aspect of cell life [25]. The modification of proteins by phosphorylation or dephosphorylation can rapidly regulate and fine-tune the protein function and activity in response to environmental signals. *Salmonella* infection has been shown to cause phosphorylation changes in many proteins in mammalian macrophages [26,27]. In a previous study, *Salmonella* infection in chicken macrophages was found to induce significant phosphorylation changes in proteins of various signaling pathways involved in metabolism and immune response using a chicken-specific kinomic immunometabolism peptide array analysis [18]. The process of aerobic glycolysis is catalyzed by the following enzymes at different steps: hexokinase, phosphoglucoisomerase, phosphofructokinase, aldolase, triosephosphate isomerase, glyceraldehyde 3-phosphate dehydrogenase, phosphoglycerate kinase, phosphoglycerate mutase, enolase, pyruvate kinase, and lactate dehydrogenase (http://www.genome.jp/kegg/pathway.html, accessed on 11 May 2023). In the present study, the peptide array analysis showed that significant phosphorylation changes in many enzymes that catalyze glycolytic pathway in the macrophage cells during *Salmonella* infection (Table 1). These changes are strong evidence indicating the shift of glycolysis in infected macrophages. However, these results only provide a general indication of significant changes in the host cell glycolysis caused by infection; the exact impact of *Salmonella* infection on glycolysis need to be further validated.

### 3.2. The Effect of Salmonella Infection on Chicken Macrophage Cell Glycolysis Varies Greatly among Salmonella Strains

**Figure 1:**
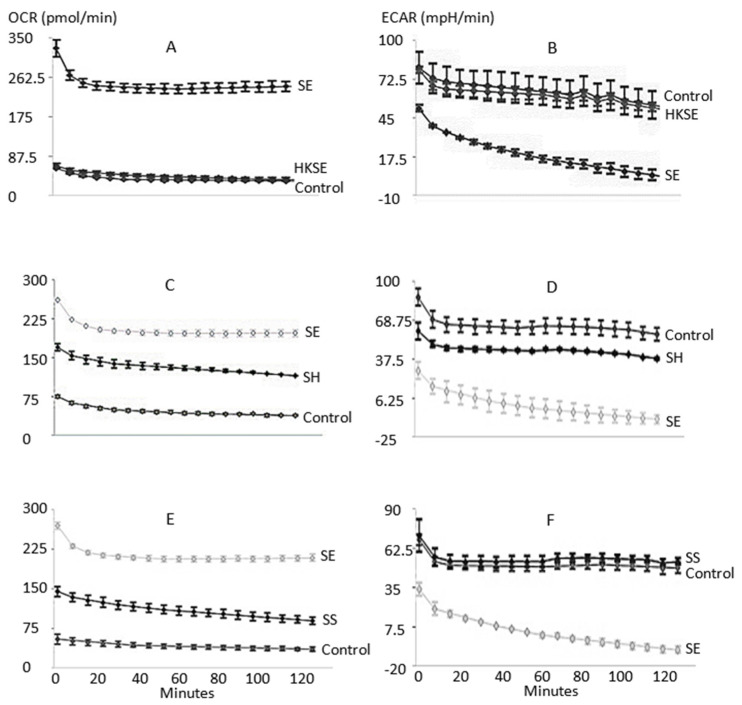
Real-time and live cell analysis of glycolysis based extracellular acidification rate (ECAR) and mitochondrial oxidative phosphorylation (OXPHOS) based on the oxygen consumption rate (OCR) were performed by the Seahorse XFp Analyzer. The effects on ECAR and OCR in macrophages of non-treated control, treated with HKSE (heat-killed *S*. Enteritidis), or infected with different *Salmonella* strains were measured. (**A**,**B**): OCR and ECAR of control, HKSE treatment, and SE (*S*. Enteritidis) infection; (**C**,**D**): OCR and ECAR of control, SE, and SH (*S.* Heidelberg) infection; and (**E**,**F**): OCR and ECAR of control, SE, and SS (*S.* Senftenberg) infection.

**Figure 2:**
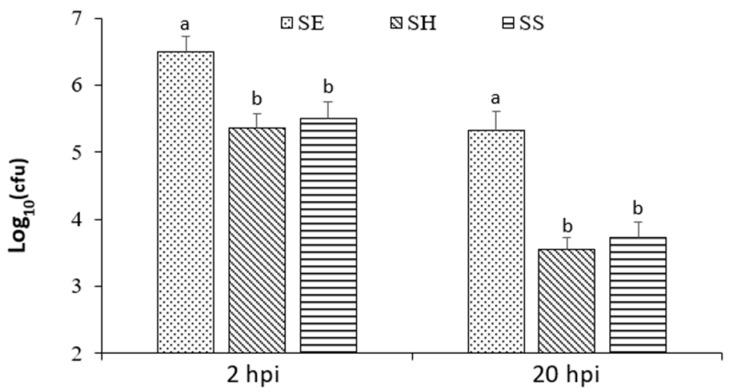
Intracellular viable *Salmonella* at 2 and 20 hpi. SE (*S*. Enteritidis), SH (*S.* Heidelberg), and SS (*S.* Senftenberg). Different letter (within each time group) indicates that the difference between the *Salmonella* strains is statistically significant (*p* ≤ 0.05).

The Agilent Seahorse analyzer measures real-time and live cell glycolysis and mitochondrial OXPHOS based on extracellular acidification rate (ECAR) and the oxygen consumption rate (OCR), respectively. In this study, the ECAR and OCR of macrophages (non-treated, treated with HKSE, or infected with different *Salmonella* strains) were measured and recorded in real-time (Figure 1). HKSE treatment of macrophages did not alter ECAR and OCR as compared to the control nontreated cells (Figure 1A,B). ECAR and OCR from macrophages infected with three *Salmonella* strains, *S*. Enteritidis, *S.* Heidelberg, and *S*. Senftenberg, were compared. Overall, all three strains elevated the OCR of infected macrophages, indicating increased OXPHOS; however, the degree of change caused by *S*. Enteritidis infection was much greater than by *S.* Heidelberg and *S*. Senftenberg (Figure 1C,E). The ECAR was greatly reduced in macrophages infected with *S*. Enteritidis and was much less affected in the cells infected with *S.* Heidelberg (Figure 1D). *S*. Senftenberg did not change the ECAR of the infected macrophages (Figure 1F). These results suggested that the impact on host cell glycolysis was *Salmonella* strain specific and most likely a contributing factor to virulence and fitness for intracellular survival within the macrophages. As shown in this study (Figure 2), *S*. Enteritidis’s ability to survive in chicken macrophages was greater than *S.* Heidelberg and *S*. Senftenberg. *Salmonella* Senftenberg is mostly an environmental strain that persists in and is frequently isolated from poultry hatching houses, farmhouses, and raw feed materials [28,29], it remains a less prevalent strain in poultry products. In this study, *S*. Senftenberg showed no effect on chicken macrophage glycolysis as measured by ECAR, which was identical to treatment with HKSE. Previously, this strain was also found to be less virulent and lacked the ability to attain systemic infection [18]. Together, the results suggest that downregulating macrophage glycolysis may contribute to increased virulence of specific *Salmonella* strains to attain a systemic infection.

### 3.3. Downregulation of Host Cell Glycolysis and Increase of M2 Polarization of Macrophages Contribute to Increased Intracellular Survival of Salmonella Strain

**Figure 3:**
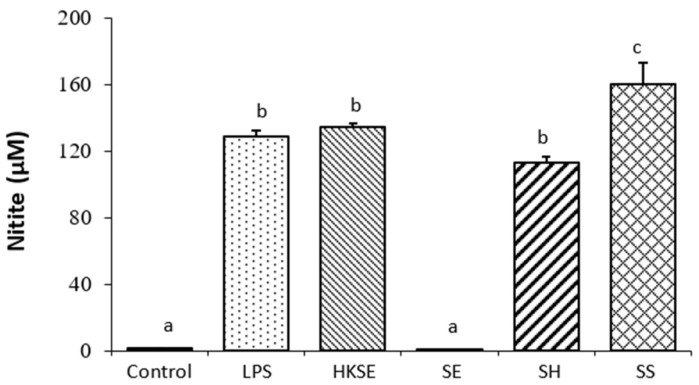
Nitrite production by activated macrophages. Macrophages were stimulated with 0.1 µg/mL of lipopolysaccharide (LPS), heat-killed *S*. Enteritidis (HKSE), or infected with three *Salmonella* strains, *S*. Enteritidis (SE), *S.* Heidelberg (SH), and *S.* Senftenberg (SS). Different letters (within each time group) indicate that the difference between the treatment groups is statistically significant (*p* ≤ 0.05).

Chicken HD-11 cell is an avian acute leukemia virus MC29 transformed macrophage cell line [19]. This cell line readily produces NO in response to stimulation by pathogen associated molecules patterns (PAMPs) [30] and *Salmonella* infection [18]. Macrophages have been described as first-line defense immune cells that are highly plastic, and their function can be changed rapidly through the process of polarization which produces pro-inflammatory (M1) and anti-inflammatory (M2) macrophages [31]. M1 macrophages display increased levels of glycolysis and reduced OXPHOS and produce high levels of NO and pro-inflammatory cytokines; M2 macrophages reduce glycolysis, enhance OXPHOS, and produce less NO [31]. In a mouse model, S. typhimurium was found to be preferentially associated with M2 macrophages at later stages of infection and intracellular replication was directly linked to the metabolic state of macrophages and the level of intracellular glucose available to bacteria [32]. Our results suggested that *Salmonella* infection promotes M2 polarization marked by reduced glycolysis and enhanced OXPHOS, except for *S*. Senftenberg. In this study, we also demonstrated that *S*. Enteritidis infection inhibited NO production, while infection with *S.* Heidelberg and *S*. Senftenberg strongly induced NO production in the macrophages (Figure 3). *S*. Senftenberg stimulated significantly higher NO production compared to LPS, HKSE, and *S*. Heidelberg, indicating that *S*. Senftenberg may promote the M1 polarization, which may explain the observation that the strain is less virulent and lacks the ability to attain systemic infection [18]. Together, these results indicated that *Salmonella* strains possessed different abilities to induce M2 macrophage polarization; *S*. Enteritidis was more effective at promoting M2 polarization than *S.* Heidelberg and *S*. Senftenberg. The strong ability to downregulate host cell glycolysis and to promote M2 polarization was associated with high intracellular survival of *S*. Enteritidis in chicken macrophages.

In summary, our study demonstrated that *Salmonella* infection altered macrophage cell glycolysis and induced M2 polarization in chicken macrophage HD-11 cells. Infection with *S*. Enteritidis and *S.* Heidelberg reduced glycolysis and enhanced OXPHOS in chicken macrophages as indicated by real-time change of ECAR and OCR. Infection with *S*. Senftenberg did not alter glycolysis, but increased OXPHOS in infected chicken macrophages. Among the three strains tested, *S*. Enteritidis was most effective in promoting M2 polarization as measured by ECAR, ORC, and NO production. The results indicated that the ability to modulate host cell glycolysis and promote M2 polarization varies depending on *Salmonella* strains. Downregulation of host cell glycolysis and increase in M2 polarization of macrophages may contribute to increased intracellular survival of *Salmonella*.

## Figures and Tables

**Table 1:** Members of glycolysis pathway showing phosphorylation change (* *p* ≤ 0.05 and ** p ≤ 0.01) from *Salmonella* Enteritidis infected microphage HD-11 cells.

Protein	p-Site Fold Δ	SE (1.5 hpi) Fold Δ	SE (3 hpi)	SE (7 hpi) Fold Δ
HK1	S299	−1.0653 **	−1.0331 **	−1.0319
	T822	1.0046	−1.0082	1.0693 **
	S828	−1.0001	−1.0273	−1.1500 **
HK2	Y304	1.0309 *	−1.0256 **	1.1142 **
	Y462	1.0180	1.0437	−1.2978 **
	T763	1.0263	−1.1318 **	−1.0723
GPI	S184	−1.1497 **	1.0014	−1.0376 *
	T108	−1.0117	−1.0091	1.1004 **
PFKL	Y664	1.0202	1.0373 *	−1.0231 *
PFKP	Y364	1.0382 **	1.0255 *	1.1579 **
ALDOB	T39	−1.0252	1.0082	1.0886 **
TPI1	Y164	−1.0990 **	1.3193 **	−1.3562 **
GAPDH	Y396	1.0803 **	1.0092	1.1195 **
	Y118	−1.0130	1.0341	−1.1206 **
PGK1	Y196	1.0548 **	−1.0782 **	−1.0290
PGM1	T496	1.0284	1.0652 **	1.0418 *
PGM2	Y565	1.0137	−1.0354 *	−1.1699 **
PGM3	S64	−1.0612 *	−1.0640 **	1.0921 **
ENO3	Y131	1.0095	1.0235	−1.0134
PKR	Y522	−1.1377 **	−1.0605 *	−1.0027
	T512	−1.0297	−1.0283	1.0893 **
PKM	Y106	1.0566 **	−1.0635 **	−1.1243 **
	Y371	1.0573 **	−1.0450 **	1.0107
	S38	1.0227	−1.0377 *	1.0781 **
LDHB	T248	−1.0025	−1.0487	−1.0962 **

Hexokinase (HK 1 and 2); Glucose-6-phosphate isomerase (GPI); 6-phosphofructokinase (PFK L and P); Fructose-1,6-bisphosphate aldolase (ALDOB); Triosephosphate isomerase 1 (TPI1); Glyceraldehyde 3-phosphate dehydrogenase (GAPDH); Phosphoglycerate kinase (PGK 1); phosphoglucomutase (PGM 1, 2, and 3); Enolase 3 (ENO3); Pyruvate kinase (PKR and PKM); Lactate dehydrogenase B (LDHB).

## Data Availability

Not applicable.
